# Head and neck single- and dual-energy CT: differences in radiation dose and image quality of 2nd and 3rd generation dual-source CT

**DOI:** 10.1259/bjr.20210069

**Published:** 2021-04-29

**Authors:** Lukas Lenga, Marvin Lange, Simon S Martin, Moritz H Albrecht, Christian Booz, Ibrahim Yel, Christophe T Arendt, Thomas J Vogl, Doris Leithner

**Affiliations:** 1 Department of Diagnostic and Interventional Radiology, University Hospital Frankfurt, Frankfurt, Germany; 2 Department of Radiology, Memorial Sloan Kettering Cancer Center, New York, NY, USA; 3 Department of Biomedical Imaging and Image-guided Therapy, Medical University of Vienna, Vienna, Austria

## Abstract

**Objectives:**

To compare radiation dose and image quality of single-energy (SECT) and dual-energy (DECT) head and neck CT examinations performed with second- and third-generation dual-source CT (DSCT) in matched patient cohorts.

**Methods:**

200 patients (mean age 55.1 ± 16.9 years) who underwent venous phase head and neck CT with a vendor-preset protocol were retrospectively divided into four equal groups (*n* = 50) matched by gender and BMI: second (Group A, SECT, 100-kV; Group B, DECT, 80/Sn140-kV), and third-generation DSCT (Group C, SECT, 100-kV; Group D, DECT, 90/Sn150-kV). Assessment of radiation dose was performed for an average scan length of 27 cm. Contrast-to-noise ratio measurements and dose-independent figure-of-merit calculations of the submandibular gland, thyroid, internal jugular vein, and common carotid artery were analyzed quantitatively. Qualitative image parameters were evaluated regarding overall image quality, artifacts and reader confidence using 5-point Likert scales.

**Results:**

Effective radiation dose (ED) was not significantly different between SECT and DECT acquisition for each scanner generation (*p* = 0.10). Significantly lower effective radiation dose (*p* < 0.01) values were observed for third-generation DSCT groups C (1.1 ± 0.2 mSv) and D (1.0 ± 0.3 mSv) compared to second-generation DSCT groups A (1.8 ± 0.1 mSv) and B (1.6 ± 0.2 mSv). Figure-of-merit/contrast-to-noise ratio analysis revealed superior results for third-generation DECT Group D compared to all other groups. Qualitative image parameters showed non-significant differences between all groups (*p* > 0.06).

**Conclusion:**

Contrast-enhanced head and neck DECT can be performed with second- and third-generation DSCT systems without radiation penalty or impaired image quality compared with SECT, while third-generation DSCT is the most dose efficient acquisition method.

**Advances in knowledge:**

Differences in radiation dose between SECT and DECT of the dose-vulnerable head and neck region using DSCT systems have not been evaluated so far. Therefore, this study directly compares radiation dose and image quality of standard SECT and DECT protocols of second- and third-generation DSCT platforms.

## Introduction

Spectral dual-energy CT (DECT) made its clinical debut in 2005 with the introduction of the first dual-source CT (DSCT) scanner.^1^ Since then, concerns regarding increased radiation exposure due to the use of a second radiation source have remained unchanged.^2^ In this context, new dose-saving techniques such as higher X-ray tube current reserves and new stellar detectors with tin filtration have been developed and implemented in third-generation DSCT.^3^ Previous studies which compared radiation dose between single-energy CT (SECT) and dual-energy DSCT of chest, abdomen and pelvis reported a dose reduction in favor of DSCT.^4–6^ Dose reduction in the head and neck region would be of particular clinical value in view of the close proximity to the orbit and the known sensitivity of the ocular lens to ionizing radiation.^7^

DECT of the head and neck region has been thoroughly evaluated in recent years, primarily focusing on oncologic^8,9^ and vascular^10^ imaging. Its utility has been demonstrated for the reduction of metal artifacts resulting from dental implants,^11^ for the detection of bone marrow edema,^12^ as well as for radiotherapy treatment planning.^13^ Moreover, DECT texture analysis coupled with novel machine learning algorithms yielded promising results, *e.g.* in the assessment of cervical lymph node pathologies.^14^ However, differences in radiation dose between SECT and DECT examinations in the dose-vulnerable head and neck region using second- and third-generation DSCT scanners have not been investigated yet.

Hence, the purpose of this study was to perform direct comparisons of radiation dose and image quality in matched patient cohorts for contrast-enhanced single-energy and dual-energy head and neck CT using second- and third-generation DSCT devices.

## Methods and materials

### Patient selection

This retrospective study was approved by our local Institutional Review Board (no. EK 311/18) with a waiver for written informed consent. Health Insurance Portability and Accountability Act criteria were met. A radiologist with more than 6 years of experience reviewed all contrast-attenuated head and neck CT scans, which had been performed between February 2013 and November 2018. Our final study population included 200 non-consecutive patients in total (100 females; mean age, 47.8 ± 19.7, range 18–88 years; 100 males; mean age, 55.1 ± 16.9, range 19–94 years). Patients were assigned to one of four study groups (Groups A–D), each containing 50 patients (25 females and 25 males, respectively). Adequate comparability of the study groups was guaranteed by matching patients by gender and body mass index (BMI). Cases with severe motion or swallowing artifacts, artifacts from dental implants and age under 18 years were excluded.

According to the vendor’s recommendations, predefined SECT and DECT examination protocols were used for all scanners. For both, second- and third-generation scanners, patients were scanned in either single-energy or dual-energy mode following protocols of ongoing IRB-approved studies at our institution (Department of Diagnostic and Interventional Radiology, University Hospital Frankfurt) at the time of examination.

### CT image acquisition

For patients assigned to Group A (SECT) and Group B (DECT), a second-generation DSCT scanner (Somatom Definition Flash, Siemens Healthineers, Forchheim, Germany) was used. Patients in Group C (SECT) and Group D (DECT) were examined with a third-generation DSCT system (Somatom Force, Siemens Healthineers, Forchheim, Germany).

For both DSCT scanner generations, X-ray tubes operated in single-energy mode at 100 kV (second generation, 235 ref. mAs; third generation 197 ref. mAs). Tube voltage and tube current settings for DECT were as follows: 80/Sn140 kV (302/151 ref. mAs) for second-generation, and 90/Sn150 kV (219/122 ref. mAs) for third-generation DSCT, respectively. Tubes operating at Sn140 kV and Sn150 kV were equipped with a tin filter for dose saving purposes (Selective Photon Shield II, Siemens Healthineers, Forchheim, Germany).

All scans were acquired in caudocranial direction from the aortopulmonary window to the frontal sinus with a pitch of 0.6. Non-ionic contrast agent (400 mg iodine/ml; Imeron, Bracco) at a dose of 1.0 ml kg^−1^ body weight with a maximum of 90 ml was injected at a flow-rate of 2 ml s^−1^, followed by a 30 ml saline flush. The scan started with a delay of 70 s after contrast agent administration in inspiratory breath hold. Real-time automatic tube current modulation software (Caredose 4D, Siemens Healthineers, Forchheim, Germany) was used in each examination to adapt tube current to patient anatomy.

### Image reconstruction

Dedicated iterative reconstruction technique was used for second-generation (Safire) and third-generation (Admire) DSCT (Siemens Healthineers, Forchheim, Germany) at a strength level of 3 of 5 with a medium smooth reconstruction kernel (B30f for second-generation; Br40 for third-generation). All data were calculated as transverse and coronal images with a slice thickness of 2.0 mm and increment of 1.0 mm. Table 1 summarizes all image acquisition data.

**Table 1.** DSCT acquisition and reconstruction parameters

	Group A	Group B	Group C	Group D
DSCT-Generation	second-Generation	second-Generation	third-Generation	third-Generation
Acquisition mode	SECT	DECT	SECT	DECT
Tube voltage	100 kV	80/Sn140 kV	100 kV	90/Sn150 kV
Tube current	235 ref. mAs	302/151 ref. mAs	197 ref. mAs	219/122 ref. mAs
Pitch	0.6	0.6	0.6	0.6
Rotation time	0.5 sec	0.5 sec	0.5 sec	0.5 sec
Collimation	128 × 0.6 mm	2 × 128×0.6 mm	192 × 0.6 mm	2 × 192×0.6 mm
Section thickness	2 mm	2 mm	2 mm	2 mm
Iterative reconstruction algorithm	Safire (strength level 3)	Safire (strength level 3)	Admire (strength level 3)	Admire (strength level 3)
Increment	1 mm	1 mm	1 mm	1 mm
Kernel	B30f	B30f	Br40	Br40
Linear-blending in dual-energy mode	–	60% 80 kV, 40% Sn140 kV	–	60% 90 kV, 40% SN150 kV
Tin filter	–	Selective Photon Shield	–	Selective Photon Shield II

DECT, Dual-energy computed tomography; DSCT, Dual-source computed tomography; SECT, Single-energy computed tomography.

### Radiation dose

Volume CT dose index (CTDI_vol_) and dose–length product (DLP) values were recorded from the patient’s protocols, which were available in our picture archiving and communication system (PACS). Mean scan acquisition length in centimeters was measured as the ratio of DLP and CTDI_vol_. As differences in scan acquisition length might have an impact on radiation exposure and to ensure reliable comparisons among the four study groups, an overall mean scan acquisition length of 27 cm for head and neck CT was used, based on all included examinations. This value was calculated by measuring and averaging the scan acquisition length of all included examinations. For both, SECT and DECT acquisition, DLP was multiplied with the International Commission on Radiological Protection conversion factor *k* for head and neck CT of 0.0058 (mSv / mGy x cm) to calculate effective dose (ED).^15^

### Quantitative image analysis

All quantitative measurements were performed by a radiologist with 5 years of experience in head and neck CT using a standard PACS workstation (Centricity 5.0, General Electric Healthcare). Circular two-dimensional regions of interest (ROIs) were drawn into the following anatomical structures to measure attenuation in Hounsfield units (HU): submandibular gland (ROI size, 30–100 mm^2^), thyroid gland (30–100 mm^2^), internal jugular vein (30–100 mm^2^), and common carotid artery (30–70 mm^2^). For each anatomical region, repeated measurements were averaged to ensure data consistency. ROIs covered the entire transaxial cross-section of the anatomic structures of interest, merely avoiding areas of focal heterogeneity originating from artery wall calcifications, oral metal implants, and swallowing or motion artifacts.

Contrast-to-noise ratio (CNR) calculations of the submandibular gland, thyroid, internal jugular vein, and common carotid artery were performed. Additional ROIs of subcutaneous fat at the level of C5 (50–120 mm^2^), and sternocleidomastoid muscle (50–120 mm^2^) were drawn to quantify image noise as standard deviation (SD) of the attenuation value measured within fat. CNR was calculated using the following formula^16,17^ :

CNR = ((HU_ROI_ – HU_muscle_)) /Noise_fat_

Comparison of quantitative image quality parameters independent of radiation dose is hampered by the fact that different tube voltage settings result in different ED values. In this context, a figure-of merit (FOM), defined as the ratio of CNR^2^ to ED, was calculated for each anatomical region at each of the four tube voltages in groups A–D. FOM values account for differences in tube voltage settings and thus comparison of CNR change independent of ED.^18^

### Qualitative image analysis

All four patient groups (A–D) were independently evaluated by three radiologists with 5–6 years of experience in head and neck CT imaging. Readers were blinded to CT acquisition protocol and scanner. DECT examinations present with characteristic field-of-view (FOV) configurations, which precludes from meaningful blinding to the technique used (*i.e.* SECT or DECT). Four readout sessions were performed, with SECT and DECT images nevertheless being read in random order in each session. A time interval of at least 2 weeks was kept between readings to reduce potential recall bias. Preset standard window settings for head and neck CT (width, 400 HU; level, 100 HU) could be freely modified. Image series were rated in terms of overall image quality, image artifacts and reader confidence using 5-point Likert scales.^19^ Scores for overall image quality (1 = non-diagnostic, 2 = poor, 3 = sufficient, 4 = good, 5 = excellent), as well as image artifacts and reader confidence (1 = major artifacts/examination non-diagnostic, 2 = severe artifacts/confidence degraded, 3 = mild artifacts/decreased confidence, 4 = minimal artifacts/confidence not affected, 5 = no artifacts perceivable/examination highly confident) were assessed individually.

### Statistical analysis

Dedicated software was applied to perform statistical analysis (MedCalc statistical software v. 19.2.0; MedCalc Software bvba).

Arithmetic means ± SDs were used for continuous variables, medians for non-continuous distributed variables. Normal distribution of data was tested using the Kolmogorov–Smirnov test. Repeated analysis of variance (ANOVA) statistics were performed in Gaussian-distributed data, whereas non-Gaussian distributed data were analyzed using the Wilcoxon signed-rank test. The impact of BMI on ED was analyzed applying Spearman correlation analysis.

5-point Likert scales were averaged and analyzed using the nonparametric Friedman test and post-hoc tests to calculate subjective overall image quality, artifacts and reader confidence. Interobserver agreement was calculated using intraclass correlation coefficient (ICC) in a two-way mixed-effects model^20^: ICC 0–0.39, poor; ICC 0.40–0.59, fair; ICC 0.60–0.74, good; ICC 0.75–1.0, excellent agreement.

In addition, differences in diagnostic image quality among the four study groups were assessed by dichotomizing the results from subjective ratings of overall image quality, image artifacts and reader confidence into unacceptable (1–2) *vs* acceptable (3–5) quality, which was indicated by the readers.^21^ Dichotomous variables were analyzed using χ^2^ or Fisher’s exact test.

*p*-values ≤ 0.05 were considered as statistically significant.

## Results

### Radiation dose

Differences between our matched study groups (A–D) in gender and BMI distribution were non-significant (both, *p* > 0.73). Comparability of study groups was confirmed by a positive correlation between ED and BMI in each group (A, r^2^ = 0.62; B, r^2^ = 0.58; C, r^2^ = 0.51; D, r^2^ = 0.54; all *p* ≤ 0.001). Mean BMI values for the study groups were as follows: Group A, 25.8 ± 3.6; Group B, 26.2 ± 3.9; Group C, 25.9 ± 3.2; and Group D, 26.2 ± 2.8.

Lowest radiation dose values were found for DLP normalized to a scan length of 27 cm for third-generation DECT Group D (185.5 ± 49.5 mGy x cm), without significant differences compared to SECT Group C (206.9 ± 23.1 mGy × cm; *p* = 0.14), but significantly different in comparison with second-generation groups A and B (*p* ≤ 0.001) (Table 2).

**Table 2.** Dosimetric parameters are given as mean ± standard deviation and range in parentheses

	Group A	Group B	Group C	Group D	*p-*value
CTDI_vol_ (mGy)	11.3 ± 0.8	10.0 ± 1.1	7.8 ± 1.4	6.9 ± 1.3	Groups A *vs* B *p* = 0.45; C *vs* D *p* = 0.15;
	(10.0–12.8)	(8.1–12.7)	(5.8–13.3)	(5.0–12.1)	All other *p* < 0.01
DLP (mGy × cm)	308.5 ± 43.0	273.7 ± 48.1	209.5 ± 38.4	188.5 ± 43.3	Groups A *vs* B *p* = 0.41; C *vs* D *p* > 0.07, B *vs* C *p* < 0.04;
	(186.0–376.2)	(203.3–411.1)	(155.8–359.9)	(136.1–326.7)	All other *p* ≤ 0.001
Effective dose (mSv)	1.8 ± 0.2	1.6 ± 0.3	1.1 ± 0.1	1.0 ± 0.2	Groups A *vs* B *p* = 0.08; C *vs* D *p* = 0.11,
	(1.2–2.3)	(1.1–2.2)	(0.9–2.1)	(0.8–1.8)	All other *p* < 0.002
Mean acquisition length (cm)	27.2 ± 3.5	27.4 ± 4.1	27.1 ± 3.4	27.2 ± 4.2	All *p* > 0.78
	(18.0–33.6)	(21.0–35.0)	(17.7–34.9)	(15.8–39.3)	
DLP 27 cm (mGy × cm)	306.2 ± 22.6	270.2 ± 30.8	206.9 ± 23.1	185.5 ± 49.5	Groups A *vs* B *p* = 0.23; C *vs* D *p* = 0.14;
	(270.5–345.9)	(218.2–341.8)	(169.9–271.6)	(116.9–329.0)	All other *p* ≤ 0.001
Effective dose 27 cm (mSv)	1.8 ± 0.1	1.6 ± 0.2	1.1 ± 0.2	1.0 ± 0.3	Groups A *vs* B and C *vs* D *p* = 0.10;
	(1.6–2.0)	(1.3–1.9)	(1.0–1.9)	(0.7–1.5)	All other *p* < 0.01

CTDI_vol_, CT volume dose index; DLP, Dose-length product.

Differences in ED were not significant between SECT and DECT acquisition for each scanner generation (*p* = 0.10). Lowest ED values for a normalized mean acquisition length were observed for third-generation DECT Group D (1.0 ± 0.3 mSv), significantly lower compared to second-generation groups A and B (*p* < 0.01) (Table 2).

In summary, all differences in ED between patients examined with second-generation DSCT (groups A and B) compared to patients examined with third-generation DSCT (groups C and D) were statistically significant (*p* ≤ 0.001). At the same time, differences between SECT and DECT acquisition did not reach statistical significance, regardless of scanner generation (*p* > 0.18) (Table 2).

### Quantitative image analysis

Third-generation DECT Group D consistently showed highest CNR values for the submandibular gland, thyroid, internal jugular vein, and common carotid artery, without a significant difference in comparison with SECT Group C (*p* > 0.06), but significantly higher compared to second-generation groups A and B (*p* < 0.04) (Table 3). Pairwise CNR comparisons of second-generation groups A and B demonstrated significantly higher values for the internal jugular vein in Group B (*p* = 0.02), while differences in all other anatomical structures did not reach statistical significance (*p* > 0.15). For all anatomical entities, CNR measurements showed no significant differences between groups B and C (*p* > 0.12).

**Table 3.** Quantitative image parameters expressed as mean ± standard deviation and range in parentheses

	Group A	Group B	Group C	Group D	*p-*value
CNR					
Submandibular gland	3.1 ± 2.9	3.0 ± 3.1	4.5 ± 3.6	6.0 ± 5.5	A *vs* B *p* = 0.70; B *vs* C *p* = 0.12; C *vs* D *p* = 0.33; all other *p* < 0.04
	(1.9–10.1)	(2.7–10.3)	(2.5–11.7)	(3.1–21.4)	
Thyroid gland	12.2 ± 6.4	13.6 ± 6.8	14.3 ± 7.7	18.6 ± 8.7	A *vs* B *p* = 0.68; B *vs* C *p* = 0.96; C *vs* D *p* = 0.06; all other *p* < 0.04
	(3.6–24.4)	(1.5–24.0)	(4.6–42.2)	(4.2–39.3)	
Internal jugular vein	13.0 ± 6.9	18.8 ± 8.4	21.8 ± 14.6	27.5 ± 12.9	B *vs* C *p* = 0.48; C *vs* D *p* = 0.11; all other *p* < 0.02
	(2.5–30.4)	(6.9–37.0)	(1.9–56.2)	(10.1–56.7)	
Common carotid artery	12.9 ± 6.2	15.3 ± 6.7	18.0 ± 12.3	23.2 ± 10.7	A *vs* B *p* = 0.15; B *vs* C *p* = 0.56; C *vs* D *p* = 0.10; all other *p* < 0.02
	(2.8–27.2)	(5.1–31.1)	(1.0–49.0)	(4.5–50.5)	
Figure of merit CNR					
Submandibular gland	11.8 ± 19.1	12.5 ± 10.6	29.2 ± 28.2	108.1 ± 70.4	A *vs* B *p* = 0.33; C *vs* D *p* = 0.22; all other *p* < 0.03
	(0.1–75.6)	(1.0–56.9)	(0.3–113.9)	(0.2–422.7)	
Thyroid gland	109.0 ± 106.7	148.3 ± 112.7	219.9 ± 281.3	424.8 ± 420.6	A *vs* B *p* = 0.38; B *vs* C *p* = 0.59; all other *p* < 0.04
	(6.2 ± 357.0)	(1.5–358.6)	(16.7–1415.1)	(13.9 ± 1525.8)	
Internal jugular vein	127.3 ± 151.9	282.4 ± 226.8	575.7 ± 716.3	907.0 ± 849.6	B *vs* C *p* = 0.13; all other *p* < 0.04
	(2.9–574.5)	(31.0–770.7)	(2.2–2675.9)	(109.5–2955.9)	
Common carotid artery	116.9 ± 108.0	178.3 ± 153.5	398.5 ± 514.9	650.1 ± 649.8	A *vs* B *p* = 0.06; B *vs* C *p* = 0.22; all other *p* < 0.04
	(3.5–418.9)	(17.0–619.2)	(0.6–2034.2)	(24.5–2966.9)	

CNR, Contrast-to-noise ratio.

Dose-independent FOM analysis revealed highest values for third-generation DECT Group D, with statistically significant differences compared to second-generation groups A and B (*p* < 0.04) (Table 3). Pairwise comparison between third-generation groups C and D showed significant differences in FOM for the thyroid, internal jugular vein, and the common carotid artery (*p* < 0.04), while differences for the submandibular gland were non-significant (*p* = 0.22). For the submandibular gland, thyroid, and common carotid artery differences between second-generation groups A and B were non-significant (*p* > 0.06), while FOM values for the internal jugular vein were significantly higher for DECT group B (*p* = 0.01). With the exception of the submandibular gland (*p* = 0.03), FOM calculations for the thyroid, internal jugular vein, and common carotid artery did not reach statistical significance between groups B and C (*p* > 0.13).

### Qualitative image analysis

**Figure 1.**
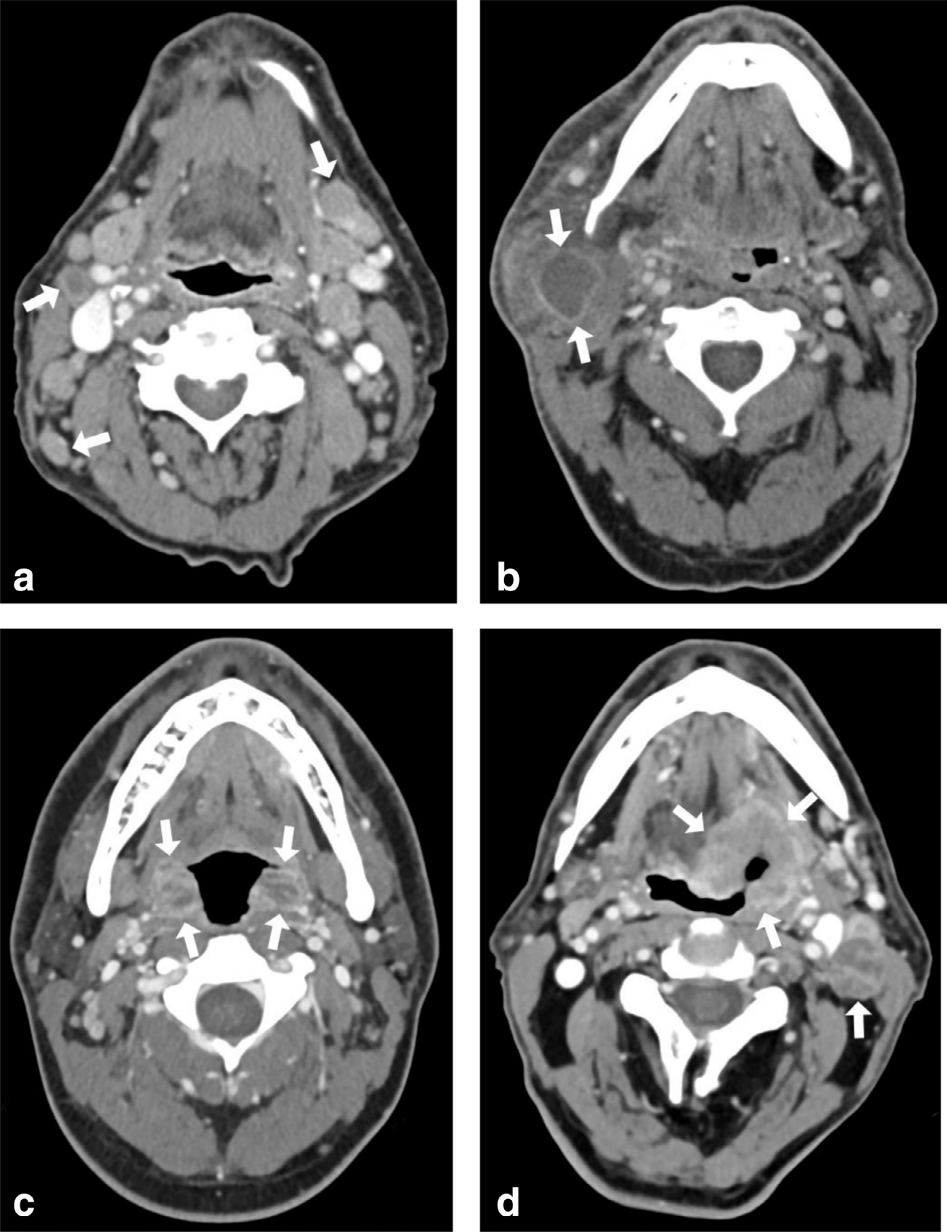
Head and neck CT examinations performed with second (**a, b**) and third-generation (c**, d**) DSCT platforms in either single-energy (a**, c**) or dual-energy technique (b**, d**). Second-generation SECT image of a 78-year-old male patient with lymphoma (arrows) and primary manifestation at the neck (a) performed at 100 kV (1.6 mSv). Image of a 63-year-old male patient with abscess of the right parotid gland (arrows) (b) obtained at 80/Sn140 kV (1.5 mSv). Third-generation SECT of the head and neck of a 24-year-old male patient with bilateral tonsilitis with abscess (arrows) (c) acquired at 100 kV (1.1 mSv). DECT image of a 77-year-old male patient with histologically proven cancer of the left tongue base and necrotizing lymph node metastasis (arrows) (1.0 mSv) obtained at 90/Sn150 kV (d) using third-generation DSCT. DECT, dual-energy CT; DSCT,dual-source CT; SECT, single-energy CT.

Overall image quality was consistently rated as good/excellent, and increased from second-generation Group A (3.3 ± 0.5) to third-generation Group D (4.4 ± 0.6), without statistically significant differences between the four groups (*p* > 0.06). Evaluation of image artifacts and reader confidence yielded good/excellent results for all groups, with highest subjective ratings for third-generation DECT Group D (4.5 ± 0.6). Lowest ratings were demonstrated for second-generation SECT Group A (3.3 ± 0.7), while average ratings for groups C and D were 3.5 ± 0.5 and 4.4 ± 0.5, respectively. Overall, differences in subjective image quality ratings did not reach statistical significance (*p* > 0.06). Figure 1 illustrates images of patients examined with second- and third-generation DSCT scanners using dedicated scan protocols of groups A–D.

Interobserver agreement was excellent for each of the four study groups in terms of overall image quality (ICC, 0.81; 95% CI, 0.73–0.86), as well as image artifacts and reader confidence (ICC, 0.82; 95% CI, 0.74–0.87). Global agreement among all readers was also excellent (ICC, 0.81; 95% CI, 0.76–0.85).

Dichotomization revealed acceptable diagnostic image quality of all examinations in groups C and D (each 50/50, 100%). In groups A 47/50 (94%) and B 49/50 (98%), examinations were considered as acceptable. Four image series were rated as being less acceptable due to artifacts from dental implants and cervical spine instrumentation. Dichotomous image evaluation showed no significant difference between the four study groups (*p* > 0.14). Table 4 outlines all qualitative image parameters.

**Table 4.** Qualitative image parameters expressed as mean ± standard deviation and range in parentheses

	Group A	Group B	Group C	Group D	*p-*value
Overall image quality	3.3 ± 0.5	3.2 ± 0.6	4.3 ± 0.5	4.4 ± 0.6	All groups *p* > 0.06
	(2–4)	(2–4)	(3–5)	(3–5)	
Image artifacts and reader confidence	3.3 ± 0.7	3.5 ± 0.5	4.4 ± 0.5	4.5 ± 0.6	All groups *p* > 0.10
	(2–5)	(3–5)	(3–5)	(3–5)	

## Discussion

The aim of our study was to compare single-energy and dual-energy head and neck CT in terms of radiation dose and image quality, using second- and third-generation DSCT systems. Our results indicate that head and neck DECT performs well with both DSCT devices, without radiation dose penalty compared to SECT acquisition. A direct comparison between second- and third-generation DSCT revealed significantly lower radiation exposure for third-generation DSCT. Quantitative FOM CNR yielded best results at third-generation DECT, while qualitative image parameters showed no significant differences between SECT and DECT acquisition. Hence, both second- and third-generation DSCT allow for routine application of head and neck CT in either single- or dual-energy mode.

Since DECT technique is based on the utilization of two X-ray beam energies, concerns about increased radiation dose at DECT examinations have initially been raised. Previous studies investigating differences in radiation dose between SECT and DECT were not able to completely eradicate these concerns, as conflicting results were reported.^2,22,23^ For instance, a phantom study performed with a second-generation DSCT system showed comparable radiation doses between SECT and DECT examinations of the chest.^2^ In this context, a study investigating radiation exposure for contrast-enhanced abdominal CT also found no differences between SECT and DECT technology using second- and third-generation DSCT devices.^16^ Few studies have evaluated differences in radiation dose between SECT and DECT in the head and neck region, demonstrating a significant dose reduction for DECT compared to SECT acquisition, while maintaining excellent image quality.^9,24^ In our institution, dual-energy mode in the head and neck region is routinely performed for the assessment of oncologic and vascular diseases. Several studies have demonstrated beneficial results without radiation penalty for the evaluation of head and neck cancer using dual-energy CT applications. For instance, the assessment of the local tumor burden can be improved using virtual monoenergetic image (VMI) reconstructions, as these allow for a more precise evaluation of lesion margins, and have the potential to reduce artifacts from oral metallic implants resulting in an improved characterization of the surrounding soft tissue.^8,11^ In addition, DECT angiography using VMI reconstructions provide enhanced carotid and intracranial artery contrast in comparison with standard images.^25^

In the present study, radiation exposure was not significantly different between SECT and DECT acquisitions for both scanner generations evaluated using preset examination protocols, with third-generation DSCT being the most dose efficient. It can be assumed that technological, hardware-based advances available in third-generation DSCT have notably contributed to the reduced radiation dose observed in our study. Third-generation DSCT operates with two novel 120-kV X-ray tubes, which significantly enhance the maximum tube power of the low kV-spectrum by increasing peak tube current up to 1300 mA. Consequently, scanning of adult patients in low-kV technique is now possible.^26^ Moreover, combination possibilities of tube voltage settings for DECT examinations were extended, ranging now from 70 kV to 150 kV for both tubes.^3^ Another substantial improvement in third-generation DSCT is based on advancements in tin pre-filtration technology. In third-generation DSCT, two selective photon shields consisting of tin filters are mounted directly in front of both X-ray tubes. Tin filtration of the high-kV tube in particular has been shown to increase mean photon energy, leading to an improved separation of the two energy spectra for better post-processing capabilities.^27^ As previously demonstrated, *e.g.* by Wichmann et al^16^ and De Cecco et al^22^, our findings can also be translated to other anatomical regions. The results of the present study are also in good accordance with previous study findings for other examinations such as CT pulmonary angiography.^28^

Quantitative FOM CNR analyses again revealed superior results for third-generation DECT. Calculation of FOM CNR as a measure of objective image quality is influenced by several factors, which are generally subdivided into acquisition (*e.g.* radiation dose) and reconstruction (*e.g.* collimation) parameters. Other aspects beside the new hardware-based developments might have contributed to improved image quality found at third-generation DSCT. New medium smooth convolution kernels available at third-generation DSCT generate images at lower image noise compared to filter kernel used at second-generation scanners, which may have influenced objective FOM CNR calculations. Moreover, the introduction of an advanced iterative reconstruction algorithm (Admire) at third-generation DSCT may have further contributed to FOM CNR. Previous investigations reported improved image quality and dose reduction for the third-generation compared to a second-generation iterative reconstruction algorithm (Safire).^29–31^ The combination and interaction of both, new reconstruction algorithm and new medium smooth filter kernel, may be potential explanations for improved quantitative image parameters found at third-generation DSCT. However, objective image quality improvements did not directly translate into improved subjective image quality ratings.

Additional dose-saving concepts in current CT generations include automated attenuation-based tube voltage selection (ATVS) and tube current modulation techniques. ATVS at third-generation DSCT has been shown to select lower tube voltages in most patients compared to second-generation DSCT ATVS, resulting in reduced radiation dose.^32^ Consequently, the application of dedicated ATVS may reduce radiation exposure at SECT acquisition. Moreover, various studies have demonstrated that routine SECT using third-generation DSCT is possible at lower tube voltages, *e.g.* 90-kVp CTA for the assessment of carotid and intracerebral vessels while simultaneously reducing radiation dose.^33^ Apart from directly lowering radiation dose as described above, DECT offers additional post-processing capabilities that have the potential to further reduce radiation exposure by calculating virtual unenhanced images.^34^ In clinical practice, the main scenarios for using dual-source single-energy mode remain cardiac imaging, as well as imaging of obese patients due to the small dual-energy FOV.

The majority of patients included in our study were diagnosed with cancer or inflammatory disease of the head and neck region. As radiation dose mainly depends on various other factors, such as tube voltage (kV), iterative reconstruction etc., we believe that case matching including other criteria (*e.g.* pathology) would not have led to different results.

We acknowledge the limitations of the present study. First, based on the retrospective study design and non-consecutive patient inclusion, selection bias cannot be excluded entirely. Second, for both scanner generations compared, we used predefined (*i.e.* unmodified vendor-provided), rather than customized, acquisition protocols for SECT and DECT, including a different medium smooth reconstruction kernel for both scanner generations (B30f for second and Br40 for third generation). New kernels of third-generation devices are designed to generate less image noise, and thus might have influenced FOM CNR calculations slightly, however, an influence on the main findings of our study seems unlikely. The use of customized protocols tailored towards further dose reduction and/or image quality enhancement may have produced slightly different results. For example, utilization of new filter kernels, dedicated iterative reconstruction algorithms, and ATVS at SECT may have further led to reduced radiation exposure and improved image quality observed at third-generation DSCT. Third, we found increased FOM CNR values for third-generation DSCT. However, increased FOM CNR does not necessarily equate into improved diagnostic image quality. To reliably evaluate diagnostic image quality, we performed a combination of both, quantitative and qualitative image analyses. Fourth, DECT examinations present with a characteristic FOV, which complicates accurate blinding during the qualitative image analysis. To overcome potential bias, we performed four readouts with sufficient time intervals between the reading sessions. Differences in qualitative image parameters were non-significant between the four groups, which indicates that the impact of FOV presentation at DECT might be minimal. Fifth, ED as a derived parameter is influenced by various factors, imprecise estimations cannot completely be avoided. However, the ‘European Working Group for Guidelines on Quality Criteria in CT’ recommends the assessment of ED for radiation exposure estimation in CT due to reliable comparisons among different modalities.^35^ ED estimations of DECT examinations are further hampered by the fact that organ-specific conversion factors for DECT acquisition do still not exist. In accordance with previous investigations, our analysis included organ-specific conversion factors that are routinely applied for SECT acquisition.^8,15^ Finally, other DECT technologies such as rapid-kV switching and dual-layer detector systems were not considered in the present study. We believe that a general transfer of our results to other technical approaches may be limited, as different technologies with underlying differences in hard- and software provided by other vendors might influence radiation dose and image quality parameters. Thus, further investigation is needed in this context.

To conclude, our findings indicate that single-energy and dual-energy head and neck CT can be performed in clinical routine without radiation penalty on second- and third-generation DSCT scanners. Differences in radiation exposure were non-significant between SECT and DECT acquisitions for both DSCT platforms, with dual-energy protocols providing similar or greater image quality compared to standard SECT. Taking functional and quantitative post-processing capabilities into account, we suggest using dual-energy mode for head and neck CT examinations irrespective of whether a second- or third-generation DSCT system is used.
